# Is There Incremental Benefit with Incremental Hearing Device Technology for Adults with Hearing Loss?

**DOI:** 10.3390/audiolres15030052

**Published:** 2025-05-06

**Authors:** Vinaya Manchaiah, Sumit Dhar, Larry Humes, Anu Sharma, Brian Taylor, De Wet Swanepoel

**Affiliations:** 1 Department of Otolaryngology-Head and Neck Surgery, University of Colorado School of Medicine, Aurora, CO 80045, USA; dewet.swanepoel@up.ac.za; 2 UCHealth Hearing and Balance, University of Colorado Hospital, Aurora, CO 80045, USA; 3 Virtual Hearing Lab, Collaborative Initiative between University of Colorado School of Medicine and University of Pretoria, Aurora, CO 80045, USA; 4 Department of Speech-Language Pathology and Audiology, University of Pretoria, Pretoria 0002, South Africa; 5 Department of Speech and Hearing, Manipal College of Health Professions, Manipal Academy of Higher Education, Manipal 576106, India; 6 The Roxelyn and Richard Pepper Department of Communication Sciences and Disorders, Northwestern University, Evanston, IL 60208, USA; s-dhar@northwestern.edu (S.D.); humes@indiana.edu (L.H.); 7 Knowles Hearing Center, Northwestern University, Evanston, IL 60208, USA; 8 Department of Speech, Language and Hearing Sciences, Indiana University, Bloomington, IN 47405, USA; 9 Department of Speech, Language, and Hearing Sciences, University of Colorado Boulder, Boulder, CO 80309, USA; anu.sharma@colorado.edu; 10 Signia, WS Audiology, Iselin, NJ 08830, USA; brian.taylor.aud@gmail.com

**Keywords:** hearing aid, hearing device, hearing device cost, technology level, accessibility

## Abstract

**Objective:** This paper reviews the current research on hearing device technology, outlines key challenges, and identifies priorities for future investigation. **Method:** This paper presents an informal narrative review of the current literature on hearing technology, supplemented by expert insights to identify key challenges and future directions. **Results:** The proliferation of direct-to-consumer (DTC) hearing devices with varied features and prices underscores the need to assess whether advanced technologies offer meaningful improvements. Understanding these incremental benefits is critical for determining the minimum technology required for optimal outcomes. The paper highlights the limitations in current clinical trials, which often suffer from selection bias, and the inadequacies of existing hearing aid outcome measures that may not capture real-life benefits. It emphasizes the need for real-world evidence and the development of assessment tools that better reflect everyday experiences. While existing research provides some insights into the potential benefits of incremental advances in hearing device technology, the evidence remains inconclusive. **Conclusions:** Addressing the cost, accessibility, and technological diversity of hearing devices is crucial to advancing hearing healthcare. Future research should prioritize the development of affordable, high-quality devices and establish comprehensive outcome measures that capture real-world benefits. A deeper understanding of these factors can lead to more accessible and effective hearing care, ultimately improving quality of life for individuals with hearing loss.

## 1. Introduction

Hearing loss is one of the most prevalent chronic conditions in older adults. According to the World Health Organization (WHO) nearly 2.5 billion people (1 in 4) are projected to have some degree of hearing loss by 2050, with at least 700 million requiring rehabilitation [1]. Despite a range of negative consequences associated with acquired hearing loss in adults, the majority of those in need of hearing rehabilitation lack access to appropriate care [2]. This issue is not confined to low- and middle-income countries (LMICs); many low-income individuals without health insurance in high-income countries also face similar challenges. For this reason, there is an urgent need for the development of low-cost hearing device solutions and effective service delivery models.

The increasing recognition of the aforementioned issues by professional organizations, government agencies, charities, and the scientific community has led to several initiatives aimed at improving access to hearing care for adults. In 2009, the National Institute on Deafness and Other Communication Disorders/National Institutes of Health (NIDCD/NIH) sponsored a working group on Accessible and Affordable Hearing Health Care for Adults with Mild to Moderate Hearing Loss [3]. This working group identified several key research questions essential for improving access and affordability in hearing care. Of these, the following two questions are particularly pertinent to hearing health outcomes and hearing device technology.

- *What is the minimal technology that will achieve success with hearing aids?* [3] (p. 5)
- *What is the difference in outcomes among very low-cost one-size-fits-all, low-cost try and select, individually programmed, trainable, and full-feature high-cost devices for varying patient population groups and for individual patients?* [3] (p. 5)

The convergence of consumer hearing device technology (e.g., earphones) with medical hearing device technology (i.e., hearing aids) over the last decade has resulted in hybrid direct-to-consumer (DTC) devices. For instance, in the United States, a new category of hearing devices, over-the-counter (OTC) hearing aids, now exists in addition to conventional prescription hearing aids. There are two types of OTC hearing aids available, pre-set [OTC-PS] and self-fitting [OTC-SF]). It should be noted here that “self-fitting” refers to technology within the device designed to adjust the electroacoustics automatically. For pre-set OTC devices, there can also be user-based self-fitting adjustments to the devices that might include user-selected adjustments of frequency-gain characteristics. There are other DTC devices available, however, aside from hearing aids. These include personal sound amplification systems (PSAPs) and hearables [4,5]. While these developments in technology have provided consumers with more options, they have also introduced confusion, raising questions about the most appropriate level of technology, including differences in costs and expected benefits across levels of technology. Thus, the critical questions posed by Donahue et al. [3] about minimum levels of technology required to achieve acceptable outcomes remain unanswered.

Given these developments and the ongoing need for improved access and affordability of hearing care, it is crucial to address the minimum levels of technology required to achieve acceptable or reasonable benefits. In this discussion paper, we discuss existing research on hearing device technology, highlighting current challenges and suggesting areas for future research. By exploring these issues, we aim to contribute to the understanding of the relationship between technology levels, personal preferences, and treatment outcomes while also informing future efforts to improve hearing healthcare accessibility and effectiveness.

## 2. Key Metrics and Considerations in Hearing Device Technology

### 2.1. Effect of Hearing Device Technology Level on Outcomes

Evidence on prescription hearing aids reported in the literature to date has not demonstrated a clear relationship between technology level (e.g., essential, standard, advanced, premium) and hearing aid outcomes such as benefits or satisfaction [6,7,8]. However, some studies have reported superior outcomes in some domains. For instance, higher technology levels have been associated with more acceptable noise levels [9], improved localization in laboratory setting [10], and greater satisfaction for speech understanding in large groups [11]. Despite these findings, most outcome measures show similar results for both basic and premium prescription hearing aid technology levels. Thus, whether the range of technological solutions available today yield different outcomes remains unknown.

It remains unknown whether the outcome measures in vogue today are responsive to the differences in outcomes, if any do exist. For instance, many users of premium technology levels report more comfort with their devices, which is not often measured using the existing hearing aid outcomes [12]. Premium technology could have less negative side effects, such as electroacoustic feedback issues, that are often not measured and reported in clinical trials in a standardized way [13]. On the other hand, some benefits observed (e.g., improved sound quality, reduced listening effort) in laboratory settings may not translate to life experiences outside the laboratory [14]. It appears that individuals who self-report a strong preference for premium technology also rate user control of the hearing aids (e.g., smartphone app), and the ability to stream phone calls and music via wireless Bluetooth to their hearing aids as highly important features [15]. Today, however, these features are not limited to premium technology and are commonly found at all technology levels. In general, the preponderance of evidence suggests that using a wide array of existing outcome measures, differences in technology levels within prescription hearing aids appear to be largely negligible for everyday outcomes.

The emergence of various DTC hearing devices, such as OTC hearing aids, PSAPs and hearables, have provided further demarcation between technology levels within hearing devices in terms of features and functionalities. Although high-quality clinical trials comparing all hearing device categories are lacking [5], some studies offer preliminary evidence for comparison between at least two categories. For instance, a recent observational study reported similar outcomes between OTC-SF hearing aids and prescription hearing aids [16]. A recent meta-analysis identified several studies where PSAPs were found to have comparable outcomes to prescription hearing aids [17]. Moreover, several studies with single-group pretest–posttest designs have reported that DTC hearing devices could have reasonable outcomes for individuals with mild to moderate hearing loss [4,5]. However, one key limitation in these studies is that the hearing devices were chosen by researchers who have often ensured the choice of good quality hearing aids within the respective categories for a study [5]. This was, in large part, because the focus was on the fitting methods or service-delivery model and not the devices themselves. This has introduced an inherent selection bias in the literature which could explain positive outcomes in a large number of studies despite wide variance in the acoustic quality of hearing devices [18,19]. In essence, these studies have provided an “existence proof” that excellent outcomes are possible with DTC devices given good-quality devices and well-developed fitting methods. This, however, does not guarantee that such outcomes will be attainable for *all* DTC devices and fitting methods.

**Figure 1:**
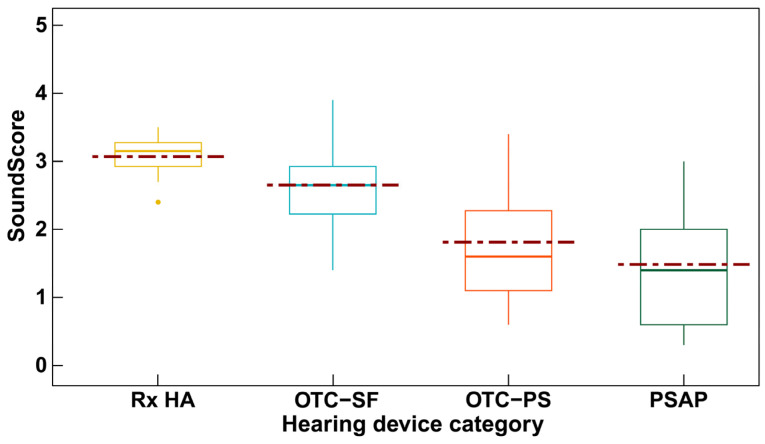
ScoundScore across hearing device categories (Rx HA = prescription hearing aids; OTC-SF = over-the counter self-fitting hearing aid; OTC-PS = over-the-counter pre-set hearing aid; PSAP = personal sound amplification system). Statistically significant differences in SoundScore were found between device categories. Reprinted from Manchaiah et al. [20].

In a recent study, the audio performance, or electroacoustic characteristics, of four categories of hearing devices were examined using a novel consumer-centric metric for hearing devices referred to as SoundScore [20]. The SoundScore can range from 0 to 5 with higher scores indicating superior audio performance. As illustrated in Figure 1, the study suggested statistically significant differences in hearing device categories based on audio performance, with prescription hearing aids having the highest ratings, PSAPs having the lowest ratings, and the OTC hearing aids falling in between. Moreover, the variability in audio performance for prescription hearing aids is much lower but the variability in audio performance for DTC devices is much larger. In other words, hearing aids irrespective of brand are likely to have good audio performance but the audio performance of DTC devices could vary depending on factors such as brand and device price [20]. Despite the absence of outcome data from clinical trials, these electroacoustic data suggest that the hearing device technology may have some relation to outcomes. On the other hand, it is well-known that a wide range of suitable electroacoustic features, such as frequency-gain responses, can yield equivalent functional outcomes, such as speech recognition in noise [21].

### 2.2. Economic Considerations in Hearing Device Accessibility

The technology level of hearing devices directly impacts their cost. For instance, in the United States, prescription hearing aids cost the consumer USD 1000 to USD 6000 per pair depending on the technology level. OTC-SF hearing aids can range between USD 600 and USD 2400 with most of them priced at around USD 800 per pair. OTC-PS devices are sold for USD 100 to USD 600 per pair and PSAPs sell for USD 20 to USD 600 per pair. While some health insurance plans may cover hearing aids fully or partially [22], this may be a sizable out-of-pocket expense for many individuals with hearing loss. This is especially true for those who are economically challenged, many of whom lack health insurance or have basic insurance with limited or no coverage for hearing aids [23,24].

What constitutes a reasonable cost for hearing devices? The answer varies widely based on factors such as geographic location, familial net worth (or wealth), income, and perceived need and benefit from the devices. For some, USD 1000 per pair may be a reasonable expense, while for others, it represents a significant financial burden. Therefore, instead of focusing solely on the device cost, it may be useful to consider the proportion of income (or savings) that users must spend on hearing devices. This approach aligns with health economics principles and better reflects the financial impact on individual families [25].

A recent clinical trial at Northwestern University exploring the effectiveness of OTC-SF hearing aids informally surveyed participants and communities about the price that they would be willing to pay out of pocket. Anecdotally, the majority considered USD 200 to be a reasonable cost, while USD 400 or more was deemed excessive. Also, a quick search on Amazon.com would suggest that most of the hearing devices being sold in large volumes are below $200. These observations suggest that a price point similar to that of high-quality headsets or earphones may be acceptable for hearing devices that can be considered as medical devices [26].

Hearing aid uptake is influenced by a range of factors, including perceived hearing difficulty, stigma, cost, and motivation [27]. Some argue that lowering the cost of hearing aids may not significantly improve hearing aid uptake [28,29]. This argument is often based on similar adoption rates in countries with different healthcare systems. For instance, in the United States where there is a lack of universal coverage for hearing aids through the health system, the hearing aid uptake rate is believed to be around 15–20% [30]. In the United Kingdom, where hearing aids are provided free of cost through the National Health Service, the hearing aid uptake rates are estimated to be around 25–30% [31], which is not considerably higher than in the United States. On the other hand, hearing aid uptake rates in many low- and middle-income countries (LMICs) are much lower with estimates between 2 and 12% [32]. These variations indicate that while cost is a barrier, hearing aid uptake is a complex issue influenced by perceived need, perceived benefit, and other factors [27]. For this reason, we believe that having a high-quality hearing device at an affordable price point can be very helpful for hearing device adoption for larger populations, especially to address the unmet need for hearing healthcare [29].

Studies investigating hearing aid use among individuals with normal audiograms provide additional insight into how cost impacts uptake. For example, Roup et al. [33] fitted hearing aids to a group of 17 individuals with self-reported hearing problems but hearing threshold levels below 25 dB HL between 250 and 8000 Hz. According to some criteria, below 25 dB HL thresholds can be considered as hearing within normal limits, although the American Speech-Language-Hearing Association (ASHA) criteria suggest a minimal hearing loss category for hearing threshold levels between 16 and 25 dB HL. Participants reported significant self-perceived hearing difficulties, particularly in challenging listening environments such as noisy backgrounds or group conversations. Although their aided benefit was similar to a control group, just three of the 17 individuals opted to purchase the devices at the end of the trial. It is concerning that only three participants indicated a willingness to purchase a hearing aid, highlighting a disconnect between subjective need and intent to seek amplification. In a double-blinded case-control study, Mealings et al. [34], fitted 27 adults between 19 and 68 years old (mean = 36 years). All participants had an average hearing threshold level below 25 dB HL. The “experimental group” were fitted with mild-gain hearing aids with advanced directional processing. The “control group” were fitted with hearing aids also, but their hearing aids were programmed to 0 dB insertion gain, with no directionality. The 0 dB insertion gain was chosen for the control group for it to be acoustically transparent—it neither amplifies nor attenuates sound at that frequency. Experimental group participants reported significantly lower levels of hearing-in-noise difficulties when they were fitted with mild-gain hearing aids compared to no device, while the placebo control group showed no difference. Additionally, participants in the experimental group reported significantly higher satisfaction with the devices than those in the placebo control group. Despite the everyday benefits reported by the experimental group (91% reported improved speech understanding over background noise), when given the option of buying the hearing aids for a purchase price of ~USD 3500 per pair, none of them agreed to this option. These studies further strengthen the argument for affordable hearing device technology.

In addition to uptake of the devices, cost may influence the retention of those devices after acquisition. In the RCT of Humes et al. [35], the three parallel service-delivery branches were stratified with about half in each branch paying USD 3600 per pair of hearing aids and half paying USD 600 per pair. The higher purchase price represented a “typical” price for prescription hearing aids at the time and the lower price was an estimate of the likely purchase price of OTC hearing aids in the near future. With rare exception, purchase price did not have a significant effect on any of the wide array of outcome measures. However, it did have a significant effect on the proportion of hearing aids returned. Among those who had paid the higher (typical) price, the return rate at 6 weeks was 22% (17 of 77), a fairly typical return rate in the U.S. at the time. However, among those who paid the lower price, the return rate was significantly lower, just 4%. Thus, purchase price significantly reduced returns after the 6-week-trial period. As with initial uptake of devices, the factors impacting retention of those devices after acquisition are also complex [27].

In addition to the out-of-pocket costs of the devices themselves, additional costs or burdens can be attributed to the need to obtain the services of a healthcare professional for prescription hearing aids. There are considerable costs in time and inconvenience associated with the need for the consumer to travel to a specific location for assistance, costs that can be largely avoided with DTC devices [36,37].

### 2.3. Precision Versus One-Size-Fits-All Approaches

Two key strategies to reduce hearing device costs are (a) embracing a one-size-fits-all approach and (b) promoting self-management. The proliferation of DTC hearing devices, such as OTC-PS hearing aids and PSAPs, has resulted in many devices designed with a one-size-fits-all philosophy. There is also growing recognition of the importance of promoting self-management within hearing healthcare [38]. Recent guidelines from the WHO suggest the use of pre-set hearing aids as well as the use of minimally trained persons as the primary facilitators for hearing aid service delivery models within low- and middle-income settings [39]. This approach contrasts sharply with the precision-based methods pursued in recent decades, which focus on high-end technology and services provided by trained professionals (e.g., audiologists). We believe that both the existing precision-based centralized and newly proposed one-size-fits-all approaches could be complementary. For individuals who have insurance coverage or can afford prescription hearing aids, a precision-based approach may offer the most personalized and effective solution. However, for those who face financial barriers or lack access to prescription hearing aids, a one-size-fits-all model may provide a practical and accessible alternative. Importantly, hearing healthcare professionals must recognize and work to reduce disparities in access and quality of care resulting from economic inequality. In this context, one-size-fits-all solutions should not be viewed as inferior but rather as a viable entry point that can promote broader access to hearing care—similar to the way over-the-counter reading glasses serve as an initial step toward more specialized vision care.

DTC hearing devices such as OTC-SF hearing aids, OTC-PS hearing aids and PSAPs can be beneficial to individuals with mild to moderate hearing loss. However, it is likely that some users may struggle with self-testing and self-fitting of the hearing devices and are likely to abandon the use if they do not get help [5]. Research is urgently needed to develop and evaluate one-size-fits-all devices and self-management-based service delivery models as a means to reduce costs and improve accessibility for all individuals who need hearing devices.

### 2.4. Meaningful Improvements with Hearing Device Intervention

The question of outcomes and hearing device technology cannot be adequately answered without considering what constitutes key outcome domains (e.g., speech-in-noise benefit, quality of life) as well as what constitutes a clinically meaningful change in these domains. While hearing aid benefit and hearing aid satisfaction are often considered as the primary outcomes of interest in the efficacy and effectiveness clinical trials on hearing aid intervention, there has not been a consensus-based approach to develop the core outcome domains that should be reported in hearing aid clinical trials. Moreover, most existing hearing aid outcomes lack data on what constitutes a minimal clinically important difference (MCID) that is needed to determine success at an individual user level [12,40].

Hearing aid interventions have been shown to exhibit placebo effects [41]. This means that the act of wearing hearing aids can lead users to report self-perceived benefits and/or perform better in laboratory settings, even if the hearing device does not provide any meaningful acoustic gain to aid their hearing loss. For example, in a double-blind placebo controlled clinical trials on hearing aids, the placebo group demonstrated some significantly improved outcomes following the use of hearing aids [3], although the benefits observed in non-placebo groups were considerably and significantly greater than those in the placebo group. Hearing aid outcomes are also subject to various confounding effects such as the narrative effect, i.e., professionals providing a positive narrative towards the hearing aid fitting process to make patients believe one approach is better than the other [42] or instructing the user to focus on positive listening experiences [43]. Although variation in fitting complexity and time do not impact outcome measures, adults may believe that longer, more complex fitting methods are superior to simpler shorter methods [44]. This may tend to bias the device user away from DTC devices with simpler fitting methods. More importantly, the need (e.g., situational versus all the time), expectations, and the effort (i.e., time and money spent) could influence the success with hearing devices at an individual level. For instance, a user might purchase an OTC-PS hearing aid or PSAP at a reasonable price (e.g., USD 200), use the device situationally, and perceive some benefit, thus considering the investment worthwhile. On the other hand, a user could spend USD 6000 on high-end premium hearing aids and would want these devices to provide optimal hearing in all situations and could be unhappy with their outcome.

Due to these factors, self-report and behavioral outcomes alone may be insufficient to assess the benefits of hearing aids and some objective measures may be necessary [45]. For example, while a user might subjectively report satisfaction, other measures might reveal areas where the device does not perform optimally. This highlights the need for a multi-faceted approach to evaluating hearing aid success, incorporating both self-report user experiences and behavioral or electrophysiological performance data.

A major challenge lies in determining what constitutes a minimal clinically important difference (MCID) or change that signifies success at both the population and individual levels. This is crucial for understanding the everyday effectiveness of different hearing device technologies. Additionally, the cost-benefit analysis of hearing devices should consider not only the financial investment but also the overall improvement in quality of life and daily functioning. By addressing these aspects, we can better understand the incremental benefits of different hearing device technologies and service delivery options to improve hearing healthcare outcomes for individuals with hearing loss.

## 3. Implications for Hearing Device Technology

Despite numerous efforts to develop low-cost hearing device solutions, devices that offer good audio performance at a price point accessible to most of the population with hearing loss is still not available in sufficient quantity, even in high-income countries such as the United States [46]. However, consumers now have access to a range of DTC hearing devices with different features and functionalities available at a range of price points. Given this diversity, there is an urgent need to understand the differential benefits associated with incremental increases in hearing device technology (and therefore, cost) for adults with hearing loss. This broader question can also help ascertain the minimum technology needed to achieve acceptable patient outcomes as well as the efficacy and effectiveness of the one-size-fits-all approach to adult hearing healthcare.

Current clinical trials may often suffer from researcher or clinician bias, where only high-quality devices are used, creating a selection bias and potentially skewing results [5]. Additionally, the wide variability in hearing devices, especially at lower price points, necessitates an emphasis on real-world evidence to inform care in the existing landscape [47]. However, designing and implementing such clinical trials can be highly challenging and can only be possible with a multidisciplinary team and will require substantial funding.

Existing hearing aid outcome measures were developed over a decade ago and may not be sensitive to small changes over repeated measurements. The outcome measures in use today may also not cover all dimensions of outcomes as highlighted in our recent systematic assessment of content validity of self-reported measures of hearing disability [48] and the measures of hearing aid benefit and satisfaction [12]. For this reason, efforts are needed to develop and validate real-world hearing aid outcome measures, such as ecological momentary assessment (EMA) technology, which can capture more ecologically valid outcomes in a more sensitive manner.

Addressing the cost, accessibility, and technological variations in hearing devices is critical for improving hearing healthcare. Future research should focus on developing affordable, high-quality hearing devices and validating comprehensive outcome measures that reflect the benefits experienced in daily life in a way that quantifies meaningful changes. By doing so, we can make substantial progress towards ensuring that hearing devices are not only accessible but also demonstrably enhance the quality of life for individuals with hearing loss. Understanding the incremental benefits of hearing device technology is essential for informing future research and policy, which can further improve hearing healthcare accessibility and effectiveness.

It is important to emphasize that this article has focused on the rapidly evolving technology landscape. It has long been recognized, however, that the devices represent just one component of a comprehensive approach to the rehabilitation of those with hearing difficulties [49,50,51]. The best devices money can buy may be of little use if support is not also provided to the user while fitting, using, and maintaining those devices. Affordable and accessible tools to assist the adult with hearing difficulties in the self-management of those difficulties on their journey to improved function are also sorely lacking [38]. This, too, is an area in need of future research and may be as important for the long-term success of adults with hearing difficulties as the device option selected at the outset of their journey.

## 4. Conclusions

Despite efforts to develop low-cost hearing devices, accessible and high-quality solutions remain insufficient even in high-income countries. The availability of various direct-to-consumer (DTC) hearing devices highlights the need to understand the incremental benefits of advanced technologies. This understanding is essential to optimize patient outcomes and assess the effectiveness of a one-size-fits-all approach. Current clinical trials and outcome measures often suffer from biases and are outdated, emphasizing the necessity for real-world evidence and the development of new, sensitive assessment tools, such as EMA technology.

While existing research provides some insights into the potential benefits of incremental advances in hearing device technology, the evidence remains inconclusive. Future research must address current limitations to determine the real-world impact of these technologies on adults with hearing loss. Prioritizing the development of affordable, high-quality hearing devices and creating comprehensive outcome measures that accurately reflect real-life benefits are crucial steps. These efforts can significantly enhance the quality of life for individuals with hearing loss by ensuring that hearing devices become both more accessible and more effective.

## Data Availability

Not applicable.
